# Sampling matters: the interplay of physiological parameters and performance over 1, 2 and 3 km in well-trained male endurance runners and sprinters

**DOI:** 10.3389/fphys.2026.1912310

**Published:** 2026-10-22

**Authors:** Yannick M. Sondermann, Oliver J. Quittmann, Simon Nolte, Marco Fuchs, Gabriel Gehlert, Tina Foitschik, Thomas Abel

**Affiliations:** 1 Institute of Sports and Sports Science, Karlsruhe Institute of Technology, Karlsruhe, Germany; 2 Institute of Movement and Neurosciences, German Sport University Cologne, Cologne, Germany; 3 Exercise Science, Coaching and Performance Enhancement (ESCAPE) Network, Cologne, Germany; 4 Institute of Exercise Training and Sport Informatics, German Sport University Cologne, Cologne, Germany

**Keywords:** exercise testing, lactate, metabolism, middle-distance, MLSS, non-oxidative, VO2max

## Abstract

**Aim:**

To examine characteristics associated with running performance in heterogeneous and endurance-only samples of well-trained male runners.

**Methods:**

Twenty well-trained (V̇O_2max_: 65.7 ± 5.5 ml·min^-1^·kg^-1^) male endurance runners (n=16) and sprinters (n=4), completed comprehensive laboratory testing and 100-m sprint as well as, 1-, 2-, and 3-km time-trials. Physiological markers included fractional utilization at maximal lactate steady-state (%V̇O_2max_), maximal fat oxidation rate (MFO), maximal lactate accumulation rate (ċLa_max_) and peak post-exercise lactate following the ramp test (La_RT_). Hierarchical regression and bivariate correlations with Benjamini-Hochberg correction were used to examine performance associations and the influence of excluding sprinters.

**Results:**

Baseline models (V̇O_2max_ + running economy) explained <40% of 1000-m and >60% in 2000- and 3000-m performance in the pooled sample. MFO increased explained variance by 15.4% at 2000 m and +14.81% at 3000 m, while %V̇O_2max_ added 11.3% at 3000 m. In the pooled sample, oxidative characteristics became more strongly associated with performance as distance increased, whereas several non-oxidative characteristics were positively associated with sprint performance and negatively associated with longer-distance performance. After excluding sprinters, associations of V̇O_2max_ and MFO with 2000- and 3000-m performance were retained, whereas associations involving ċLa_max_ and La_RT_ were attenuated. ASR remained negatively associated with 3000-m performance in both samples.

**Conclusion:**

In well-trained male runners, associations between physiological characteristics and running performance depend on event distance and sample composition. Oxidative characteristics were particularly relevant to 2000- and 3000-m performance, whereas the apparent relevance of several non-oxidative characteristics partly reflected the inclusion of sprinters. These sampling-related findings require confirmation in larger, balanced cohorts.

## 1 Introduction

Understanding the key determinants of performance is essential for practitioners and coaches aiming to optimize performance outcomes. Although each sport places unique psychological, cognitive, tactical, biomechanical, and physiological demands on athletes, endurance running is primarily governed by physiological factors. Classical deterministic models identified maximal oxygen uptake (V̇O_2max_), its fractional utilization (%V̇O_2max_) at the maximal lactate steady state (MLSS), and running economy (RE) as the principal determinants of endurance running performance (Jones, 2023).

For short endurance running, i.e. middle-distance (800–3000 m), the non-oxidative (anaerobic) metabolism has been proposed as a complementary determinant. This is plausible given the increasing reliance on substrate-level phosphorylation as event duration decreases (Hargreaves and Spriet, 2020). In support of such a proposal, non-oxidative capacity has recently been highlighted as a key factor to enable elite female runners to break the 4-min mile barrier (Osborne et al., 2025). Furthermore, concepts such as the maximal lactate accumulation rate (ċLa_max_) and anaerobic speed reserve (ASR) have sparked renewed interest concerning the non-oxidative metabolism in the context of endurance exercise (Quittmann, 2026; Sandford et al., 2021).

Research on elite middle-distance athletes underscores the importance of oxidative metabolism but rarely provides clear associations between performance and non-oxidative measures (Brandon, 1995; Kenney and Hodgson, 1985; Schnabel and Kindermann, 1983; Svedenhag and Sjödin, 2008). Sandford et al. did not report a direct link between performance and ASR but observed that elite 800-m specialists with superior 400-m performance exhibited higher ASR than those with faster 1500-m personal bests (Sandford et al., 2019). Similarly, Thron et al. demonstrated that sprinters possess higher ċLa_max_ and maximal sprinting speed than middle-distance runners (Thron et al., 2024). Duffield et al. did observe a direct relationship between performance and non-oxidative metabolism and found that greater non-oxidative contribution was associated with faster 800-m performance (Duffield et al., 2007).

Although some evidence suggests that the non-oxidative metabolism is relevant for short-endurance performance, few studies have examined performance across multiple distances while simultaneously assessing oxidative and non-oxidative determinants. Moreover, the predictive value of physiological characteristics (e.g. V̇O_2max_) seems to depend on the underlying sample as correlations to performance are typically higher in heterogeneous groups (Bassett and Howley, 2000). Prior research seldomly included athletes from diverse competitive backgrounds, let alone sprinters, whose divergent physiological characteristics may influence performance. Therefore, the present study aims to investigate the relationship of multiple oxidative and non-oxidative measures with running performance over 1-, 2-, and 3-km in a heterogeneous sample of well-trained male sprinters and endurance runners.

## 2 Methods

### 2.1 Participants

Sample size estimation using G*Power software (v3.1.9.6 *a priori* power analysis with linear multiple regression, fixed model, R^2^ increase, total number of predictors = 6, tested predictors = 4, α = 5%, 1–β = 95%) was performed according to statistical guidelines (Abt et al., 2025, Abt et al., 2020) and indicated that this study would require a minimum of 25 participants. The effect size for the power analysis (R^2^ = 50%, f^2^ = 1) was set since previous research found that typical physiological determinants explain 40–60% of the variation in middle-distance performance, and account for approximately 80% of the variance in 3- and 5-km time trials (Blagrove et al., 2019; Quittmann et al., 2022). Participants were recruited until the required sample size was attained. Due to time constraints or acute sickness, 4 of the initial 29 participants, did not complete the full study.

N = 25 well-trained male athletes (25.1 ± 4.8 years, V̇O_2max_ = 65.6 ± 5.5 ml·min^-1^·kg^-1^, RE = 221 ± 11 ml·kg^-1^·km^-1^) volunteered to participate in this study (De Pauw et al., 2013). We recruited participants from local sport clubs and at the university if they trained at least three times a week for their respective distance. To ensure a variety of physiological profiles and performances, participants ranged from sprinters (200 and 400 m) to ultra-marathon runners (> 42.195 km). The participants received a medical check-up and could only enrol in the study if cleared. Given the heterogeneous training background of the included athletes, females were not recruited to control for additional variance. All procedures received institutional ethics approval (No. 092/2021) according to the Declaration of Helsinki. Before the investigation, participants were personally informed about the aims, procedures, and potential risks of this study, and gave their written consent.

### 2.2 Experimental design

The participants performed various exercise tests to determine the individual metabolic profile and endurance performances during a three-week period (**Figure 1**). A minimum of 48 hours between test days was provided to minimize fatigue. On their first laboratory visit, the participants were informed about the procedures, received the medical check-up and underwent a ten-site skinfold thickness measurement (Harpenden Skinfold Caliper, Baty Int., West Sussex, United Kingdom) to determine their body fat percentage (Pařízková, 1977). Afterwards, the participants performed an incremental test on a motorized treadmill (saturn 300/100, h/p/cosmos sports & medical GmbH, Nussdorf-Traunstein, Germany). Two days later, a 100-m all-out sprint test was performed on a straight 100-m indoor track followed by one hour of rest and a ramp test until subjective exhaustion on a treadmill. In the second test week, several constant load tests were performed on a treadmill to determine %V̇O_2max_ and velocity at MLSS (v_MLSS_). During the third and final test week, the participants performed TT over 1000, 2000, 3000 m in randomized order on an outdoor track.

**Figure 1:**
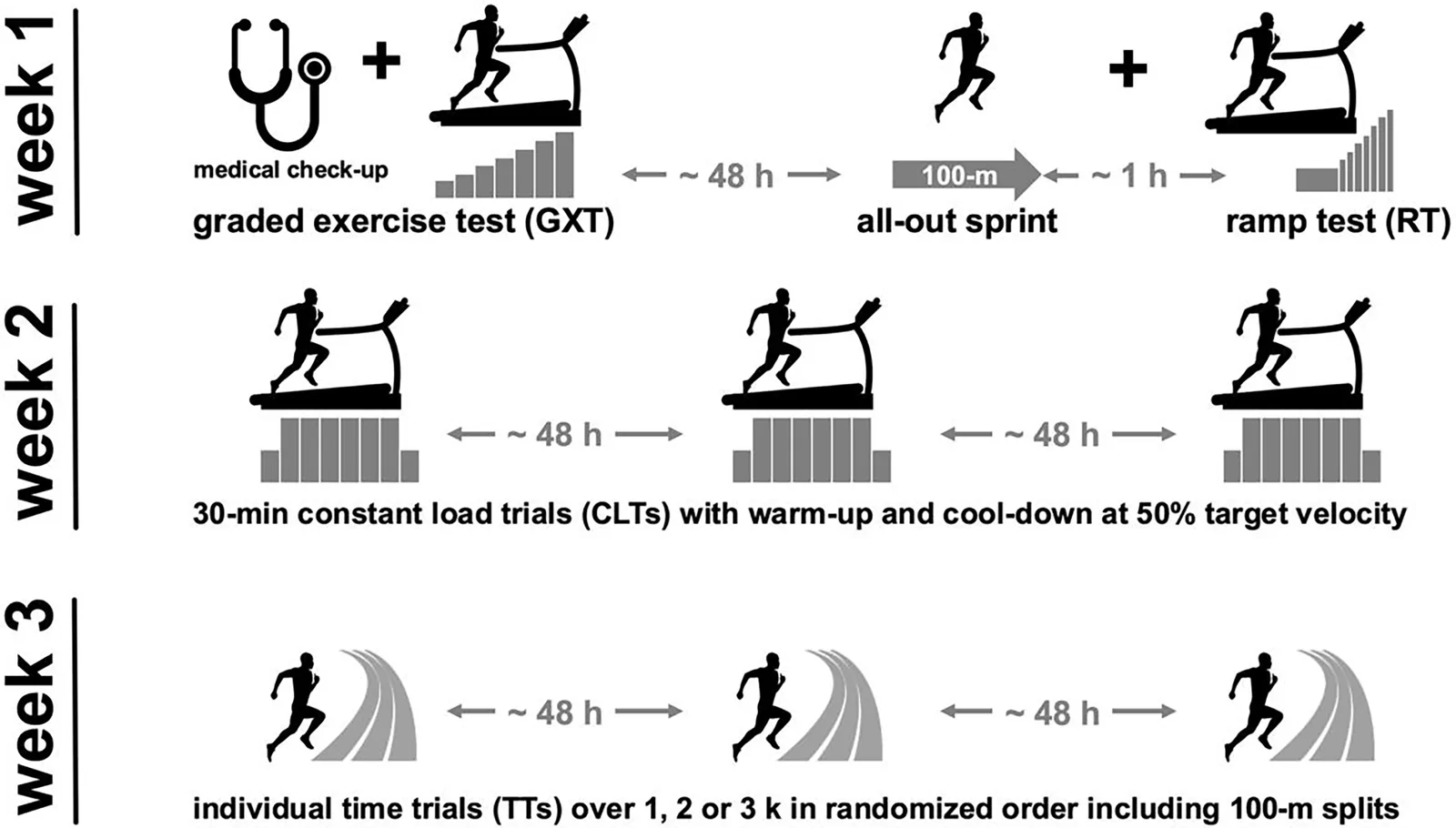
Schematic representation of the experimental design.

The incremental step test, 100-m all-out sprint and ramp test protocol were performed according to a preliminary study (Quittmann et al., 2022). However, in this study we collected post-exercise lactate concentration after the ramp test immediately after as well as 3 and 6 min afterwards to ensure a more valid measure of the highest lactate concentration (La_RT_). La_RT_ was unavailable for one participant, due to a measurement error. Consequently, bivariate analysis involving La_RT_ were based on 19 participants. To ensure comparability across models, all hierarchical regression analysis were conducted using the same 19 complete cases.

The intensity for the first constant velocity test was determined as the velocity associated with four mmol·l^-1^ blood lactate during the incremental step test. The participants ran at target velocity for 30 min interspersed with 30 s resting periods every five minutes for blood sampling. Gas exchange analysis were performed throughout the test. The criterion of lactate steady-state was defined as an increase in lactate concentration during the final 20 min of no more than 1 mmol·l^-1^ (Jones et al., 2019). If the steady-state criterion was met, target velocity in the following trial increased by 0.1 m·s^-1^. In the case of exceeding the criterion, target velocity in the following trial decreased by 0.1 m·s^-1^. If a participant was unable to finish a trial due to premature fatigue, the intensity for the next test decreased by 0.2 m·s^-1^. MLSS was defined as the highest intensity at which the steady-state criterion was met. RE and %V̇O_2max_ were based on the mean V̇O_2_ derived from the final 2 min of each 5min section within the last 20 min of the constant velocity trials.

Time trials (TTs) over 1000, 2000, 3000 m were completed on an outdoor 400-m tartan track in randomized order. A standardized warm-up of about 20 min preceded each trial (Quittmann et al., 2022). The participants completed the trials without other participants on the track. They were instructed to achieve the best time possible over the respective distance and received verbal encouragement throughout. Testing was conducted in temperatures between 15–25 °C and only when local meteorological reports indicated wind speeds below 3 m·s^-1^.

### 2.3 Calibration procedures

All measurement devices were maintained and calibrated in accordance with the manufacturers’ instructions. Before data collection, the blood lactate analyzer and cardiopulmonary exercise testing (CPET) system were serviced and calibrated by their respective manufacturers, while treadmill belt speed and gradient were calibrated by an authorized manufacturer technician. Before each CPET-based test, the flow sensor was calibrated using a 3-L syringe (Hans Rudolph Inc., Shawnee, KS, USA), and the gas analyzers were calibrated using a certified reference gas (16% O**_2_**; 5% CO**_2_**) and measurement of ambient air. The blood lactate analyzer was calibrated hourly using the manufacturer-provided calibration standard, and its measurement accuracy was verified using control samples.

### 2.4 Data processing

Raw data was collected in Excel (version 16.59). Further processing and statistical analysis were carried out with RStudio (version 2023.06.1 + 524). Gas exchange data was processed in compliance with recent guidelines (Nolte et al., 2023). Complete access to data processing and statistical analysis is available via GitHub.

### 2.5 Statistical Analysis

Descriptive data are presented as mean and standard deviation (x̄ ± SD). Normality of data was assessed based on the majority decision of five independent and blinded reviewers rating Q-Q plots of residuals (Aldor-Noiman et al., 2013). The authors deliberately chose to use visual inspection of normality rather than statistical testing in order to a) observe a whole pattern rather than a single number and b) to be able to identify how badly the data align with normality assumption when compared to a dichotomous answer. Pearson’s correlation coefficients (r) and Fisher *z*-transformed 95% confidence intervals were calculated for normally distributed variables. Otherwise, Spearman’s rank correlation coefficients (r_s_) and confidence intervals with a corrected standard error according to Fieller were calculated. The 32 endurance-only performance correlations included in the sensitivity analysis were adjusted as a separate exploratory family. Raw *p* values, Benjamini–Hochberg-adjusted *q* values, correlation coefficients, and 95% confidence intervals are provided in Appendix 1.

Hierarchical regression was used to examine whether selected physiological characteristics explained additional variance in 1000-, 2000-, and 3000-m performance beyond a prespecified baseline model. The baseline model contained V̇O_2max_ and RE, as these variables are considered the major predictors of endurance performance (Mougin et al., 2026). To avoid overfitting of the regression models with respect to sample size, only one additional parameter was entered into these (three-parameter) models (Babyak, 2004). Additional physiological characteristics – namely %V̇O_2max_, MFO, ċLa_max_ and La_RT_ – were entered separately to evaluate their incremental explanatory contribution. Multicollinearity was assessed by the maximum of variance inflation Factor (VIF).

Because the number of complete cases was lower than anticipated, a sensitivity analysis was conducted for the final hierarchical regression structure. With 19 complete cases, three total predictors, one tested predictor, α = 0.05, and 80% power, the minimum detectable incremental effect was *f*² = 0.53. Based on the observed baseline R² values, this corresponded to approximate detectable increases in R² of 0.21, 0.12, and 0.10 for the 1000-, 2000-, and 3000-m models, respectively.

## 3 Results

Twenty-five completed laboratory testing and twenty participants completed all three time-trials. Participant characteristics and physiological outcomes for the pooled and specialized groups are presented in **Table 1**. Mean sprint and time-trial performances were 11.8 ± 0.8 s, 2:53 ± 0:11 min, 6:23 ± 0:25 min and 10:06 ± 0:46 min for 100, 1000, 2000 and 3000 m, respectively.

**Table 1:** Descriptive statistics of test parameters displayed for all participants and respective athlete groups.

Variables		All runners (N = 20)	Endurance runners (n = 16)	Sprinters (n = 4)
body length	[cm]	180.6 ± 5.9	180.9 ± 6.0	179.3 ± 6.2
body mass	[kg]	68.4 ± 5.6	67.6 ± 4.8	72.0 ± 8.0
body fat	[%]	11.2 ± 2.1	11.1 ± 2.1	11.63 ± 2.7
V̇O_2max_	[ml·min^-1^·kg^-1^]	65.7 ± 5.5	66.9 ± 5.3	60.8 ± 3.9
RE	[ml·min^-1^·km^-1^]	220.6 ± 10.6	216.9 ± 7.70	235.5 ± 7.2
%V̇O_2max_	[%]	83.7 ± 4.2	84.0 ± 3.8	82.7 ± 6.0
MFO	[g·min^-1^]	0.42 ± 0.11	0.44 ± 0.12	0.37 ± 0.02
v_MLSS_	[m·s^-1^]	4.17 ± 0.48	4.32 ± 0.40	3.55 ± 0.13
CV	[m·s^-1^]	4.68 ± 0.40	4.80 ± 0.35	4.19 ± 0.15
ċLa_max_	[mmol·l^-1^·s^-1^]	0.73 ± 0.16	0.67 ± 0.09	0.97 ± 0.15
La_RT_	[mmol·l^-1^]	8.99 ± 1.60	8.34 ± 0.97	11.44 ± 0.86
D’	[m·s^-1^]	197 ± 57	179 ± 46	268 ± 42
ASR	[m·s^-1^]	2.27 ± 0.65	2.00 ± 0.31	3.34 ± 0.53

Values are expressed as mean ± standard deviation.

ASR, aerobic speed reserve; ċLa_max_, maximal lactate accumulation rate; CV, critical velocity; D’, curvature constant; La_RT_, maximal lactate concentration following the ramp test protocol; MLSS, maximal lactate steady-state; RE, running economy at maximal lactate steady-state; V̇O_2max_, maximal oxygen uptake; %V̇O_2max_, fractional utilization of maximal oxygen uptake at maximal lactate steady-state.

The baseline model (V̇O_2max_ + RE) of hierarchical regression accounted for progressively more variance as distance increased from 1000 to 3000 m (**Table 2**). All models included the same n = 19 complete cases and a maximum of three predictors. Multicollinearity was low, with maximum VIF values <1.7. Adjusted R^2^ was substantially lower in the 1000-m (<40%) when compared to 2000-m and 3000-m (>60%). Whereas the 1000-m model did not significantly improve by the above-mentioned physiological parameters, 2000-m prediction improved by MFO (R^2^ +15.5%, p=0.004) and the 3000-m model by MFO (+14.1%, p=0.001) and %V̇O_2max_ (+11.3%, p=0.006). The significant increments observed for MFO in the 2000- and 3000-m models and for %V̇O_2max_ in the 3000-m model all exceeded these the minimum detectable increases in R^2^, which were approximately 0.21, 0.12, and 0.10 for the 1000-, 2000-, and 3000-m models, respectively.

**Table 2:** Hierarchical regression analysis for running performance.

Distance (m)	Model	R^2^	adj. R^2^	ΔR^2^	ΔF	Δβ [95% CI]	p	VIF_max_
1000	V̇O_2max_ + RE	0.397	0.322					1.14
	+ %V̇O_2max_	0.397	0.277	0.001	0.01	+0.02 [-0.41, 0.46]	.911	1.18
	+ MFO	0.433	0.320	0.036	0.96	+0.21 [-0.25, 0.68]	.342	1.37
	+ ċLa_max_	0.516	0.419	0.119	3.68	+0.45 [-0.05, 0.94]	.074	1.69
	+ La_RT_	0.497	0.396	0.100	2.97	+0.38 [-0.09, 0.85]	.105	1.46
2000	V̇O_2max_ + RE	0.644	0.600					1.14
	+ %V̇O_2max_	0.699	0.638	0.055	2.71	+0.24 [-0.07, 0.55]	.120	1.18
	+ MFO	0.798	0.758	0.154	11.42	+0.44 [0.16, 0.71]	.004**	1.37
	+ ċLa_max_	0.653	0.583	0.008	0.37	+0.12 [-0.30, 0.54]	.554	1.69
	+ La_RT_	0.667	0.601	0.023	1.04	+0.18 [-0.20, 0.57]	.324	1.46
3000	V̇O_2max_ + RE	0.719	0.684					1.14
	+ %V̇O_2max_	0.832	0.798	0.113	10.09	+0.34 [0.11, 0.57]	.006**	1.18
	+ MFO	0.860	0.832	0.141	15.08	+0.42 [0.19, 0.65]	.001***	1.37
	+ ċLa_max_	0.720	0.664	0.001	0.06	-0.04 [-0.42, 0.33]	.807	1.69
	+ La_RT_	0.720	0.664	0.001	0.03	-0.03 [-0.38, 0.32]	.868	1.46

Baseline model included V̇O_2max_ and RE as these physiological parameters have been found to be the most important predictors of endurance running performance. The results display results for the single addition of different physiological variables to the baseline model.

p, the p-value from the nested-model F-test; adj. R^2^, adjusted determination coefficient (explained variance by the model); ċLa_max_, maximal lactate accumulation rate; La_RT_, maximal post-exercise lactate concertation following the ramp test; MFO, maximal fat oxidation rate; R^2^, determination coefficient (explained variance by the model); RE, running economy (at maximal lactate steady-state); VIF_max_, maximum variance inflation factor; V̇O_2max_, maximal oxygen uptake; %V̇O_2max_, fractional utilization of maximal oxygen uptake (at maximal lactate steady-state); Δβ, chance in standardized correlation coefficient, ΔF, change in variance ratio; ΔR^2^, change in determination coefficient (explained variance by the model). ** (p<0.01), *** (p<0.001). Bold font was used for all statistically significant p values for visual reasons

In the pooled sample, V̇O_2max_ was positively associated with performance over 1000, 2000, and 3000 m, with progressively larger coefficients at longer distances (r=0.677, r=0.806, r=0.843, respectively; p ≤ 0.001), but was not associated (r=-0.061, p=0.797) with 100-m performance (**Figure 2**). A similar distance-dependent pattern was found for v_MLSS_ (r_s_=0.618, r=0.850, r_s_=0.915 and r_s_=-0.331, respectively) and MFO (r_s_=0.502, r_s_=0.731 and r_s_=0.752, respectively). RE was negatively associated with 2000- and 3000-m velocity (r=-0.528, p=0.017 and r=-0.545, p=0.013, respectively), but not with 1000-m velocity (r=-0.341, p=0.142). %V̇O_2max_ was not significantly associated with performance over any investigated distance.

**Figure 2:**
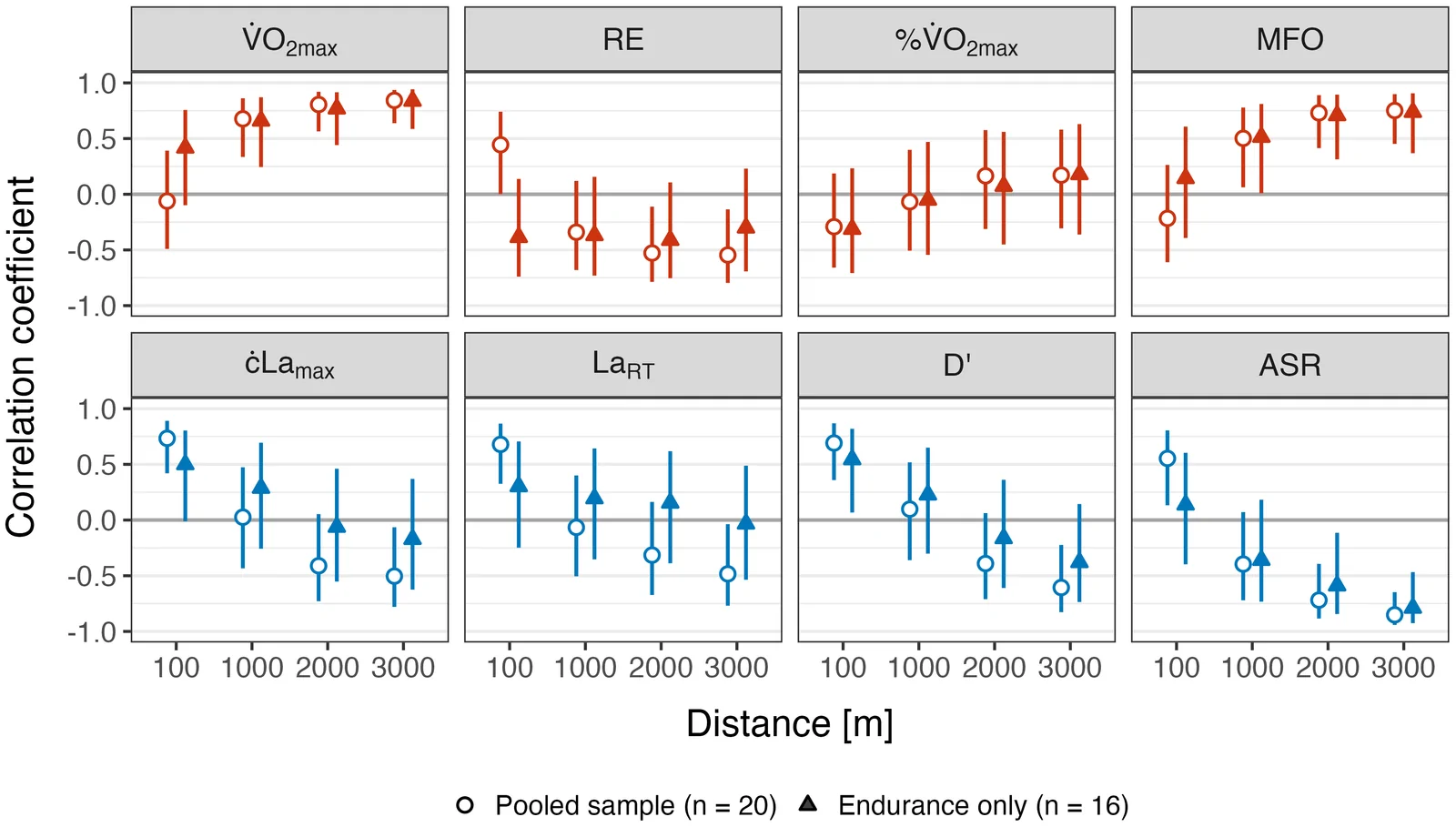
Correlations between running performance and physiological characteristics. Correlations coefficients and confidence intervals presented for oxidative (V̇O_2max_, RE, %V̇O_2max_, MFO) and non-oxidative variables (ċLa_max_, La_RT_, D’, ASR). Open circles represent the pooled sample (n = 20), and filled triangles represent the endurance-only sample (n = 16). For analysis involving La_RT_, n = 19 in the pooled sample and n = 15 in the endurance-only sample. Statistical significance was evaluated using Benjamini–Hochberg-adjusted q-values, which are reported in Appendix 2. ASR, anaerobic speed reserve; ċLa_max_, maximal lactate accumulation rate; D’ curvature constant (‘non-oxidative’ capacity); La_RT_, maximal post-exercise lactate concentration following the ramp test; MFO, maximal at oxidation rate; RE, running economy (at maximal lactate steady-state); V̇O_2max_, maximal oxygen uptake; %V̇O_2max_, fractional utilization of maximal oxygen uptake (at maximal lactate steady-state). ASR, anaerobic speed reserve; ċLamax, maximal lactate accumulation rate; D’ curvature constant (‘non-oxidative’ capacity); LaRT, maximal post-exercise lactate concentration following the ramp test; MFO, maximal at oxidation rate; RE, running economy (at maximal lactate steady-state); V̇O2max, maximal oxygen uptake; %V̇O2max, fractional utilization of maximal oxygen uptake (at maximal lactate steady-state).

Markers related to the non-oxidative metabolism – ċLa_max_, La_RT_, D’ and ASR – were positively associated with 100-m velocity in the pooled sample (r_s_=0.734, r=0.679, r=0.692 and r_s_=0.553, respectively). Conversely, these parameters were negatively associated with 3000-m velocity (r_s_=-0.504, r=-0.483, r=-0.60 and r_s_=-0.851, respectively). Complete correlation coefficients and 95% confidence intervals are provided in Appendix 1.

Sensitivity analysis were conducted after excluding the four sprinters with no significant group-parameter interaction observed (Appendix 1). However, of the 18 statistically significant associations observed in the pooled sample, 10 remained significant in the endurance-only sample. The largest differences when comparing regressions between pooled and endurance-only subsample were observed for V̇O_2max_, RE and MFO in the 100-m, as well as ċLa_max_ and La_RT_ in the 2000-m and 3000-m (**Figure 3**, Appendix 1). The associations of V̇O_2max_ with 1000-, 2000-, and 3000-m performance remained similar in direction and magnitude (r=0.660, p=0.005; r=0.769, p<0.001; and r=0.838, p<0.001, respectively). MFO also remained positively associated with performance at all three distances (r_s_=0.515, p=0.041; r_s_=0.709, p=0.002 and r_s_=0.738, p=0.001, respectively). ASR remained negatively associated with 2000- and 3000-m performance (r_s_=-0.588, p=0.017; and r_s_=-0.788, p<0.001, respectively).

**Figure 3:**
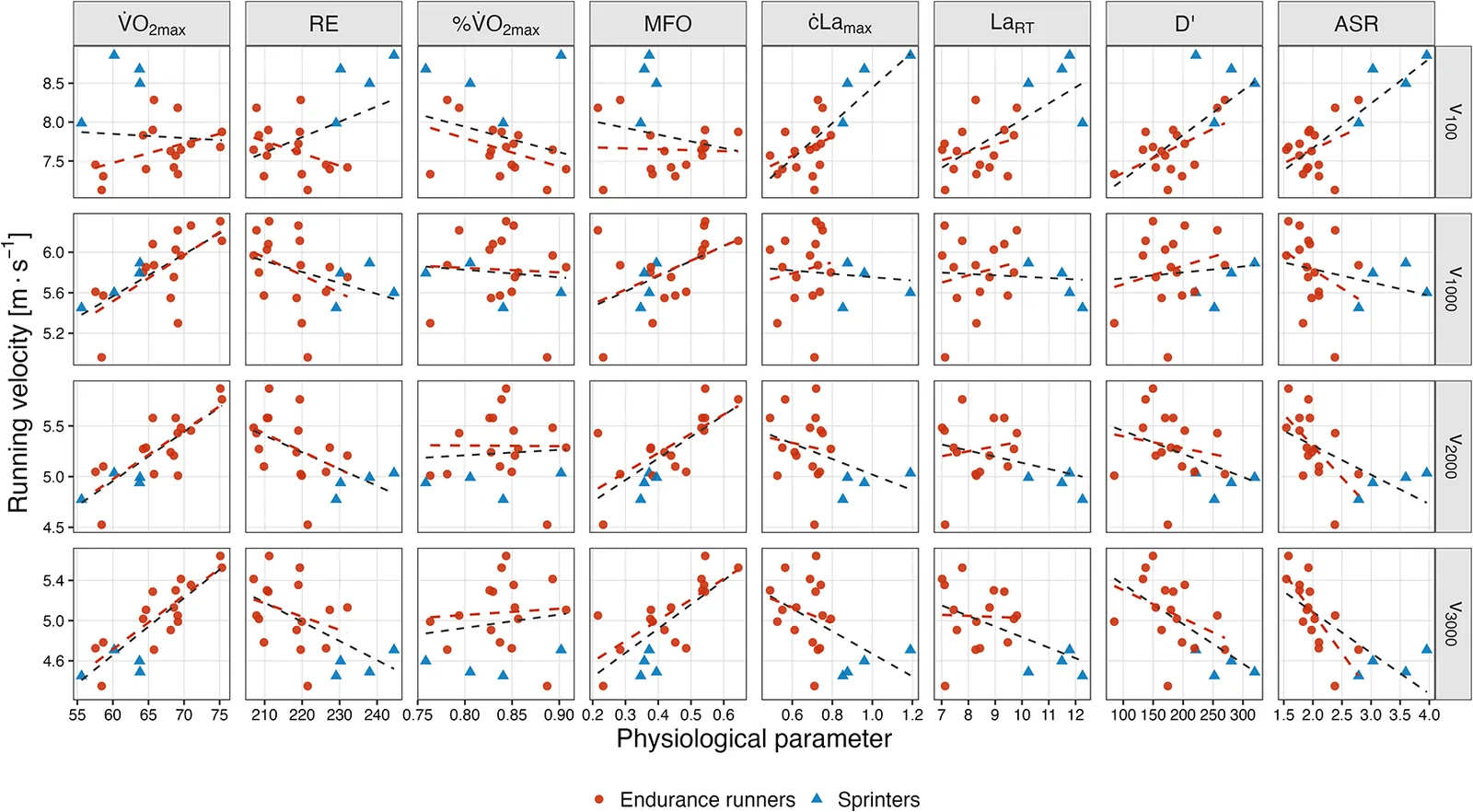
Scatter plots for running velocity and physiological variables. Sprinters (turquoise) are indicated by triangles and endurance runners (orange) by dots. The dashed line represents the regression line. ASR, anaerobic speed reserve; ċLa_max_, maximal lactate accumulation rate; D’ curvature constant (‘non-oxidative’ capacity); La_RT_, maximal post-exercise lactate concentration following the ramp test; MFO, maximal at oxidation rate; RE, running economy (at maximal lactate steady-state); V̇O_2max_, maximal oxygen uptake; %V̇O_2max_, fractional utilization of maximal oxygen uptake (at maximal lactate steady-state).

In contrast, several pooled associations with longer-distance performance were attenuated in the endurance-only sample. This included RE at 2000 m (r=-0.411, p=0.114) and 3000 m (r=-0.300, p=0.259), as well as ċLa_max_ (r_s_=-0.171, p=0.528), La_RT_ (r=-0.032, p=0.909), and D′ (r=-0.379, p=0.148) at 3000 m. Group-adjusted and exploratory parameter-by-group interaction models are presented in Appendix 1.

Relationships among physiological characteristics are shown for the pooled sample (**Figure 4A**) and for endurance runners only (**Figure 4B**). %V̇O_2max_ was not significantly associated with any other characteristic in either sample. In the pooled sample, V̇O_2max_ and MFO were positively associated with v_MLSS_ and CV, whereas RE was negatively associated with these two variables. Markers related to the non-oxidative metabolism – ċLa_max_, La_RT_, D’ and ASR – were generally positively interrelated and negatively associated with V̇O_2max_, MFO, CV and v_MLSS_.

**Figure 4:**
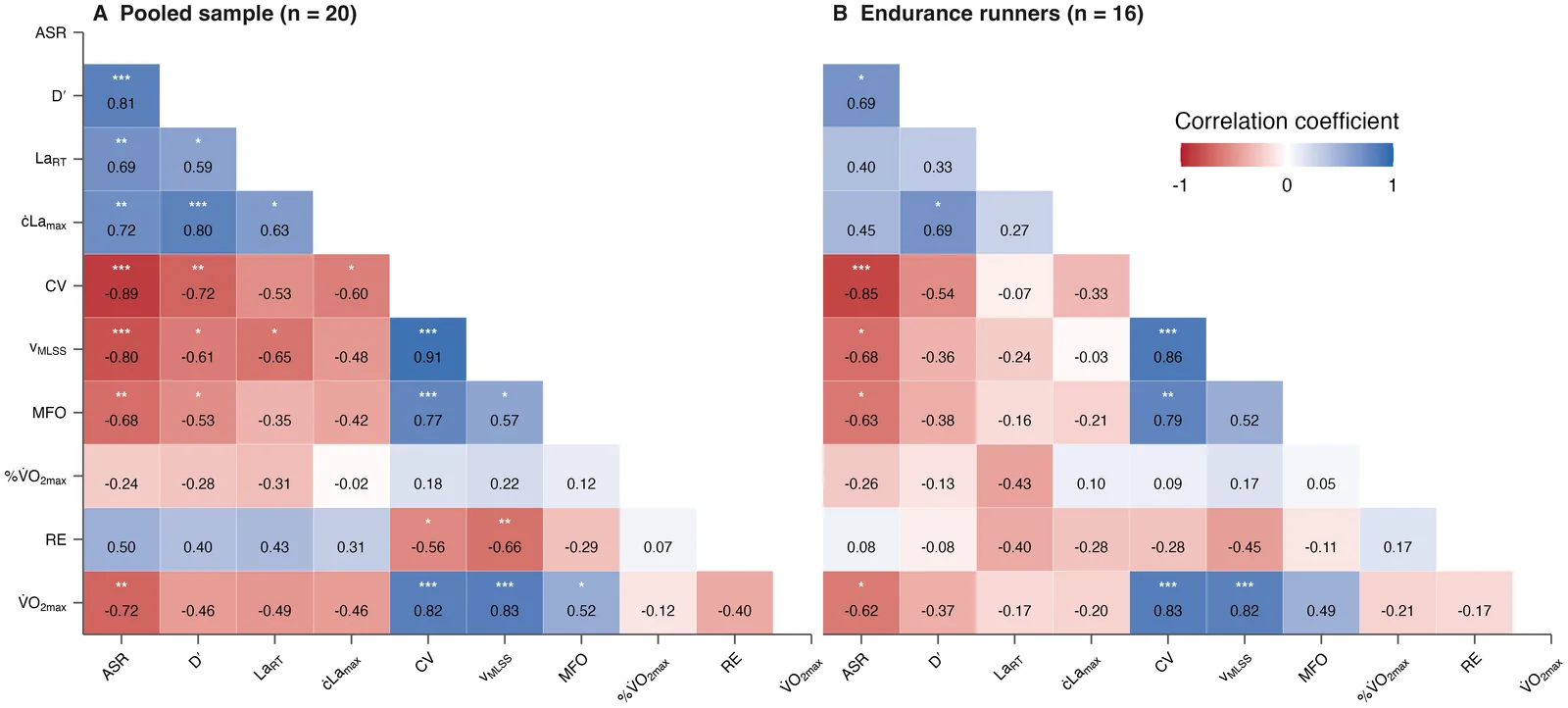
Correlation matrices of physiological and performance-derived characteristics. **(A)** presents the pooled sample of runners who completed all time trials (*n* = 20), and **(B)** presents the endurance-only sample (*n* = 16). Red tiles indicate negative correlations and blue tiles indicate positive correlations. Significance indicators are based on Benjamini–Hochberg-adjusted *q*-values: *q < 0.05, **q < 0.01, and ***q < 0.001. ASR, anaerobic speed reserve; ċLamax, maximal lactate accumulation rate; D’ curvature constant (‘non-oxidative’ capacity); LaRT, maximal post-exercise lactate concentration following the ramp test; MFO, maximal at oxidation rate; RE, running economy (at maximal lactate steady-state); V̇O_2max_, maximal oxygen uptake; %V̇O_2max_, fractional utilization of maximal oxygen uptake (at maximal lactate steady-state).

## 4 Discussion

This study in well-trained male athletes, highlights that predictors of running performance are specific to event distance and in parts affected by (sub-)sampling. We found that the baseline model (V̇O_2max_ + RE) accounts for <40% of performance variance in the 1000 m and >60% in the 2000 m and 3000 m. While MFO seemed to significantly improve performance prediction in 2000- and 3000-m running, no significant benefits were found in 1000-m running. Whereas V̇O_2max_, v_MLSS_ and RE were significantly associated with endurance performance (1000- 3000 m), non-oxidative markers – like ċLa_max_, La_RT_, D’ and ASR – were positively related to 100- and 1000-m velocity. The effect of sampling on endurance performance predictors was most dominant for lactate-derived measures of non-oxidative power (ċLa_max_) and capacity (La_RT_). A total of six out of 14 significant correlations among physiological/metabolic characteristics became attenuated when analysing endurance runners only.

Predictions of exercise/running performance by means of physiological parameters have a long tradition in exercise science (Billat, 1996). Since the pioneering work of Archibald Viviane Hill on V̇O_2max_, RE and critical velocity (Bassett, 2002) to the introduction of lactate threshold (Wackerhage et al., 2022) and the Joyner model (Joyner, 1991) up to today, many parameters, models and concepts have been introduced to explain the processes underlying exercise performance. However, the predictive value of a physiological characteristic is not ‘pure’ but depends on the underlying sample. Even though V̇O_2max_ is considered most predictive parameter of endurance performance, its predictive power is largely attenuated in a group characterized by homogeneously high V̇O_2max_ (Bassett and Howley, 2000). Similarly, our study aimed to assess this effect for a whole metabolic profile which is affected by distance and sampling.

V̇O_2max_ is considered to represent maximum oxidative power or the upper limit of oxidative phosphorylation. As such, it was found to be a physiological determinant of endurance performance from the 800-meters to ultra-marathon events (Watanabe et al., 2024; Pastor et al., 2022). Since the TTs performed in this study are within this range, it seems logical that V̇O_2max_ was associated with 1-, 2- and 3-km performance. We observed that correlations were higher and confidence intervals lower for longer distances, which seems to coincide with the increasing energy contribution of oxidative metabolism over time (Hargreaves and Spriet, 2020). Comparative studies have also observed higher V̇O_2max_ in long- rather than middle-distance runners (Brandon, 1995; Schnabel and Kindermann, 1983; Svedenhag and Sjödin, 2008; Watanabe et al., 2024). Interestingly, previous studies in competitive 800-m runners found a substantially higher performance explanation (R^2^ = 63%) when compared to our findings in the 1000-m including RE (R^2^ = 39%) (Watanabe et al., 2024). Given the high demands on both oxidative and non-oxidative metabolism over these short endurance events and the heterogeneous running backgrounds in this study, we speculate that good performances can be accomplished with specialization in either of these systems. For this reason, V̇O_2max_ might be less predictive for 1000-m in a heterogeneous sample. However, the correlation between 1000-m performance and V̇O_2max_ was similar in the endurance-only group. As such, the V̇O_2max_ does not seem to lose its predictive power when using a heterogeneous sample that includes sprinters. In summary, we confirm that V̇O_2max_ is one of the best physiological determinants of short endurance performance (1–3 km). However, its predictive value seems to increase for longer events with possibly more oxidative energy contribution.

In contrast to V̇O_2max_, recent research concerning %V̇O_2max_ at MLSS or lactate threshold (LT) reports only a marginal independent contribution to endurance-performance proxies (Johansen et al., 2025; Mougin et al., 2026; Sondermann et al., 2026). In accordance with our findings, previous research found no or rather small contributions of %V̇O_2max_ on 5000-m performance (Quittmann et al., 2022; Støa et al., 2010). Similar conclusions were highlighted from 1500-m to marathon running performance (Tanji et al., 2017; Gordon et al., 2017). Hence, we argue that %V̇O_2max_ does not seem to be a performance related parameter on its own, but rather reflects v_MLSS_ as defined by V̇O_2max_, %V̇O_2max_ and running economy (Joyner, 1991; Sondermann et al., 2026). The incremental contribution of %V̇O_2max_ to the 3000-m model likely reflects its complementary role alongside V̇O_2max_ and RE in describing MLSS velocity rather than an independent association with performance. Two possible sources of inconsistences in %V̇O_2max_ arise from the determination of both V̇O_2max_ and the exact (submaximal) intensity (MLSS, LT, etc.). To ensure sturdy measurement and avoid extrapolation assumptions, we measured %V̇O_2max_ within the constant velocity tests for MLSS determination. In accordance with recent research, we argue that %V̇O_2max_ is not a useful determinant of short endurance performance (Sondermann et al., 2026). Future studies need to provide guidelines for determining %V̇O_2max_ and discuss its value for assessing endurance performance.

The concept of MLSS aims to reflect a state of metabolic homeostasis and has shown to be a strong predictor of endurance performance over various distances (Svedenhag and Sjödin, 2008; Gordon et al., 2017). Accordingly, v_MLSS_ explained the most variance for all TT distances in this study. Since MLSS represents a physiological state that possibly combines several parameters in one, we do not consider this to be a physiological but rather performance parameter. Interestingly, v_MLSS_ was related negatively to non-oxidative measures in the pooled sample, which could indicate that athletes of superior endurance performance might lack non-oxidative capabilities. However, it is questionable whether this is a causal relationship or reflective of the lack of endurance training within the recruited sprinters.

RE has been shown to be a relevant performance variable in running and can distinguish between runners with similar V̇O_2max_ but dissimilar performance level (Barnes and Kilding, 2015). We found that runners with superior RE performed better in 2000- and 3000-m TTs, but not in the 1000-m trial. However, RE and its predictive value was affected by (sub-)sampling. Especially in the 2000-m and 3000-m TTs, RE correlated more strongly with performance when including sprinters. This deviation could be due to the assumption that sprinters typically have a higher abundance of that Type II muscle fibers (Costill et al., 1976), which are considered to be less efficient (Stienen et al., 1996). However, since we did not determine muscle fiber typology, we can just speculate about the underlying mechanisms. Ultimately, the predictive value of RE seems to be higher for longer distances than very short endurance running performances (≤ 3 min). This could be a notion that shorter distances require athletes to be effective rather than efficient, which has also been proposed elsewhere recently (Osborne et al., 2025). In summary, RE seems to be of particular importance in events ≥2 km.

MFO reflects the capacity to oxidize fat during submaximal exercise and is commonly associated with metabolic flexibility and prolonged endurance performance (Chávez-Guevara et al., 2022). A higher MFO may support longer-duration exercise by reducing carbohydrate utilization and delaying glycogen depletion (Maunder et al., 2018). However, fat oxidation contributes minimally at intensities at or above MLSS (Achten et al., 2003), making a direct energetic contribution to 2000- and 3000-m performance unlikely. Nevertheless, MFO remained associated with 2000- and 3000-m performance after Benjamini-Hochberg correction and when added to the hierarchical models, increased the explained variance by 15.4% and 14.1%, respectively, beyond V̇O_2max_ and RE.

Rather than indicating a direct contribution of fat oxidation during these time trials, MFO may represent a broader endurance-trained phenotype. Endurance training, particularly greater training volume, is associated with mitochondrial adaptations, including an enhanced oxidative metabolism and greater utilization of fatty acids during submaximal exercise. Accordingly, higher MFO has been observed in athletes with greater endurance-training exposure and performance levels (Maunder et al., 2018). Although speculative, MFO may therefore provide complementary information about mitochondrial oxidative capacity, metabolic flexibility, and accumulated endurance-training adaptations that is not fully captured by V̇O_2max_ or RE. However, the present correlational design cannot determine whether MFO itself contributes to short-endurance performance or whether both characteristics reflect common adaptations to endurance training. Future studies should examine whether the association between MFO and short-endurance performance persists after accounting for training volume and direct markers of mitochondrial adaptation.

The theoretical concept of ċLa_max_ was introduced in 1986 to explain the metabolic origin of anaerobic threshold (Mader and Heck, 1986). It is regarded to be an indicator of glycolytic (non-oxidative) power and seems to be the glycolytic equivalent of V̇O_2max_ (Wackerhage et al., 2025). The ċLa_max_ was already found to correlate with sprint performance measures in cycling, running, swimming and rowing (Quittmann, 2026). This study confirms these previous findings since ċLa_max_ correlated positively with 100-m sprint performance. Since glycolytic metabolism is responsible for providing higher rates of energy for a short period of time and contributes more in shorter exercises of high-intensity, these associations appear logical (Hargreaves and Spriet, 2020). However, ċLa_max_ was highly affected by sampling as it substantially changed its predictive value in the endurance-only group. Whereas moderate negative associations were found with 2000- and 3000-m performance (r= –0.41 and r= –0.50, respectively) in the pooled sample, these associations were rather small (r<0.20) in endurance runners. Interestingly, ċLa_max_ tended to be positively associated with 1000-m performance (r=0.29) in endurance runners, while this relationship was close to zero (r=0.03) in the pooled sample. Accordingly, we hypothesise that sprinters and endurance athletes are able to perform well in events of ∼3 min by mobilising non-oxidative and oxidative pathways, respectively. Even though this might be affected by heterogeneous training background, we argue that our findings illustrate the ‘bigger picture’ of glycolytic contributions in different running events (Hargreaves and Spriet, 2020; Bex et al., 2016). Since lactate is considered a physiological bridge between substrate-level and oxidative phosphorylation (Brooks et al., 2021), the ability to change its concentration by means of ċLa_max_ requires context for recommendations. Hence, the lack of significant correlations might not imply that ċLa_max_ is irrelevant in short endurance running events, but that there is just no linear association (Quittmann, 2026). Differences and similarities of metabolic profiles across various running specialists have recently been illustrated by Thron et al. (Thron et al., 2024). It was demonstrated that ċLa_max_ correlates with ASR, which was in accordance with the findings of this study. Further, finishing kick or last lap performance from 1500 to 5000-m were shown to positively correlate with ċLa_max_ and muscle typology (Quittmann et al., 2022; Bellinger et al., 2020), indicating that non-oxidative characteristics might have an influence on tactical behavior in competition running. Although faster sprint times require a higher power output by the work muscles (Morin et al., 2012), some researchers propose that sprint performance is primarily limited by musculoskeletal force of the lower extremity rather than metabolic power (Bundle and Weyand, 2012).

In contrast to glycolytic/non-oxidative power, non-oxidative capacity refers to a finite amount of energy that can be derived from non-oxidative energy sources (Noordhof et al., 2013). Several concepts have been developed to quantify non-oxidative capacity. Though their validity has been questioned, D’ and La_RT_ have been considered measurements of non-oxidative capacity previously (Duffield et al., 2007; Noordhof et al., 2013). In accordance with previous findings in a similarly heterogeneous group of participants, we demonstrated that V̇O_2max_ negatively correlated with lactic capacity (LT_RT_) (Schnabel and Kindermann, 1983). Despite previous studies that identified non-oxidative capacity as a determinant of 800-m running performance, we could not replicate this for the 1-km TT (Tanji et al., 2018; Lacour et al., 1990; Billat, 1996). While maximal post-exercise lactate concentrations following exhaustion, i.e. La_RT_, is a rather physiological approach to quantify lactic capacity, D’ and ASR are more practically developed and are defined as the distance that can (theoretically) be covered above critical velocity and the difference between maximal sprint and maximal aerobic speed, respectively (Sandford et al., 2019; Jones et al., 2019). While the concepts differ substantially, we demonstrated significantly positive correlations between these three non-oxidative parameters, which is in accordance to previous research (Thron et al., 2024). Interestingly, La_RT_ tended to correlate negatively with endurance performance in the pooled sample, but positively in endurance runners. Inferior endurance performance and higher lactic capacity observed in sprinters, could be a possible explanation for this finding. As such, a final conclusion about the relevance of lactic capacity utilization in short endurance events cannot be drawn. In contrast, ASR showed the clearest practical potential among the sprint-related characteristics. Previous research supports its use for profiling athletes from the 400 m through the 800–1500-m events (Sandford et al., 2019; Thron et al., 2024; Rappelt et al., 2025). Our findings extend this perspective by showing that ASR remained associated with 2000- and 3000-m performance among endurance runners. Therefore, ASR might be valuable for practitioners to characterize the athletes’ balance between sprint and endurance speed across the continuum from long-sprint to short-endurance. In conclusion, these findings emphasize the specificity of relationships between physiological profiling and running performance by means of event distance and (sub-)sampling. We highlight that running events lasting ∼3 min are less predictive than events ≥6 min, as the latter rely more on proxies of oxidative metabolism.

### 4.1 Limitations

To the best of our knowledge, this is the first study to extensively assess sprinters using measures related to the oxidative and non-oxidative metabolism, maximal lactate steady-state and multiple exhausting time trials within one single investigation. We aimed to recruit equal samples of long distance runners, (ultra–)marathoners and sprinters (n=10), but encountered particular difficulties recruiting trained sprinters to complete the endurance tests. Consequently, the sample was unbalanced, with only four sprinters, and a separate sprint-only analysis was not considered appropriate. The recruited sprinters demonstrated superior sprint performance, higher values for non-oxidative measures (ċLa_max_, D’, ASR) and showcased lower endurance performance than the endurance runners. Whether their lower endurance performance was attributable to higher substrate-level phosphorylation, less endurance-training exposure, or other specialization-related factors cannot be determined from the present findings. Given the small sprint subgroup, estimates concerning athlete specialization may be sensitive to individual observations and should be interpreted cautiously. These sampling-related findings should therefore be considered exploratory and confirmed in larger cohorts with more balanced representation of sprint- and endurance-trained athletes.

Although the *a priori* power analysis indicated a required sample of 25 participants, only 20 completed all three time trials, and the hierarchical regression analysis were conducted using 19 complete cases. A sensitivity analysis indicated that the final models had 80% power to detect an incremental effect of *f*^2^ ≥ 0.53, corresponding to approximate increases in R² of 0.21, 0.12, and 0.10 for the 1000-, 2000-, and 3000-m models, respectively. The significant increments observed for MFO in the 2000- and 3000-m models and for %V̇O_2max_ in the 3000-m model exceeded these values. Thus, the sensitivity analysis did not alter the principal regression findings. Nevertheless, the models were adequately powered only to detect large incremental effects, meaning that smaller contributions from the remaining physiological characteristics may have gone undetected.

Another limitation is the inclusion of male athletes only. Consequently, the findings cannot be generalized to female runners. Previous evidence indicates potential sex-related differences in substrate utilization, muscle-fiber distribution and non-oxidative capabilities (Boisseau and Isacco, 2021; Nuzzo, 2023; Micke et al., 2025), potentially causing further heterogeneity beyond athlete specialization. Hence, we encourage future studies to replicate the present analysis in adequately powered samples of female athletes and consider relevant female-specific factors, including menstrual-cycle phase where appropriate.

The large number of bivariate correlations increased the risk of false-positive findings (Hochberg and Benjamini, 2006). We therefore applied the Benjamini–Hochberg procedure across the 122 correlations presented in **Figures 2**, **4** and report the resulting adjusted q values in Appendix 1. Of the 59 associations with an unadjusted p <.05, 47 remained significant after correction. Although the correction reduced the number of statistically significant findings, the principal results and overall interpretation were retained. Nevertheless, the analysis remain exploratory, and the observed relationships should be examined in larger and more balanced samples.

Finally, some of the tested parameters should be interpreted with care. For example, maximum sprinting speed (a variable needed to calculate ASR) was not assessed by using high resolution equipment (e. g. laser velocity guard), but rather by the average sprint time. Hence, we slightly underestimate the participants’ true ASR. Even though ċLa_max_ was found to be highly reliable in running, it is still lacking proper validation (Quittmann, 2026). Similarly, lactate-based and gas-exchange measurement have been questioned concerning their valid representation of non-oxidative capacity and power (Noordhof et al., 2013). To compensate for this lack of validity, we assessed multiple non-oxidative parameters which demonstrated close inter-relationships. Future studies should therefore replicate these findings using larger and more diverse samples and more direct or independently validated measures.

### 4.2 Practical applications

Based on our findings in well-trained male runners, we recommend that physiological profiles should be interpreted with respect to the runners’ population and event demands. In a heterogeneous sample, non-oxidative or sprint-related characteristics differentiated sprinters from endurance-oriented profiles but mostly showed limited evidence of distinguishing 1000–3000-m performance within endurance runners. Out of these variables, ASR seems to be the most promising and remains sensible in hetero- and homogenous cohorts alike. With respect to lactate-derived measures of non-oxidative power and capacity, practitioners should aim for individualized goal-values rather than maximizing these as with V̇O_2max_. This accounts to lactic power (ċLa_max_), that might enable superior short sprint performance and lactic capacity (La_RT_), that may improve long sprint and endspurt performance, while middle-distance benefits remain inconclusive. Despite weak associations with performance, proxies of non-oxidative metabolism might still be reflective of athlete specialization, tactical capabilities, or specific race demands that are not captured by mean time-trial velocity. Even though middle-distance races are (almost) exclusively performed at carbohydrate metabolism, MFO and Fat_max_ may provide useful information for prescribing easy runs in ‘zone 2’ and the overall metabolic reliance on substrates – irrespective of athlete specialization.

## 5 Conclusion

In conclusion, among well-trained male runners, the relationships between physiological characteristics and running performance were specific to event distance and partly influenced by athlete sampling. We highlight that the measured physiological characteristics were less predictive for running performance lasting ~3 min compared to performances lasting ≥6 min. In contrast to performances ≥6 min, which were mainly associated with oxidative measures, sprint performance was related to proxies of non-oxidative capacity and power. As these non-oxidative measures seem to switch their predictive value of endurance performance by means of the underlying sample, future studies need to verify the additional benefit of these capabilities with a more balanced and greater sample.

## Data Availability

The original contributions presented in the study are included in the article/**Supplementary Material**. Further inquiries can be directed to the corresponding authors. Complete access to data processing and statistical analysis is available via GitHub (https://github.com/yannickmaxschwarz/SimProRun).
