# Supplementary material for: Sampling matters: the interplay of physiological parameters and performance over 1, 2 and 3 km in well-trained male endurance runners and sprinters
Source: Front Physiol. 2026 Oct 22;17:1912310. doi: 10.3389/fphys.2026.1912310 (PMC13735571; doi:10.3389/fphys.2026.1912310)
Supplement: Supplementary file 1 [file SupplementaryFile2.docx]

**Appendix 1 – Correlations between physiological parameters and performance (average running speed)**

| **Parameter** | **Method** | **Distance** | **Pooled (n=20)** | | | **Endurance-only (n=16)** | | | **Δ r** | **Group–adjusted** | **Group × Parameter** |
| --- | --- | --- | --- | --- | --- | --- | --- | --- | --- | --- | --- |
|  |  | **[m]** | **r [95% CI]** | **p** | **q** | **r [95% CI]** | **p** | **q** |  | **β [95% CI]** | **β [95% CI]** |
| V̇O_2max_ | Pearson | 100 | -0.06 [-0.49, 0.39] | 0.797 | 0.824 | +0.42 [-0.10, 0.76] | 0.107 | 0.303 | 0.48 | +0.34 [-0.01, 0.68] | 0.48 [–0.70, 1.65] |
|  |  | 1000 | +0.68 [0.34, 0.86]** | 0.001 | 0.005 | +0.66 [0.24, 0.87]* | 0.005 | 0.029 | -0.02 | +0.75 [0.34, 1.16]** | 0.06 [–1.39, 1.50] |
|  |  | 2000 | +0.81 [0.56, 0.92]*** | < 0.001 | < 0.001 | +0.77 [0.44, 0.92]** | < 0.001 | 0.005 | -0.04 | +0.75 [0.42, 1.09]*** | –0.44 [–1.59, 0.70] |
|  |  | 3000 | +0.84 [0.64, 0.94]*** | < 0.001 | < 0.001 | +0.84 [0.59, 0.94]** | < 0.001 | 0.002 | 0 | +0.73 [0.45, 1.01]*** | –0.67 [–1.59, 0.24] |
| RE | Pearson | 100 | +0.44 [0.00, 0.74] | 0.050 | 0.103 | -0.38 [-0.74, 0.14] | 0.141 | 0.338 | -0.83 | -0.18 [-0.66, 0.30] | 1.15 [–0.11, 2.41] |
|  |  | 1000 | -0.34 [-0.68, 0.12] | 0.142 | 0.235 | -0.37 [-0.73, 0.16] | 0.161 | 0.344 | -0.03 | -0.45 [-1.13, 0.24] | 0.69 [–1.27, 2.65] |
|  |  | 2000 | -0.53 [-0.79, -0.11]* | 0.017 | 0.044 | -0.41 [-0.75, 0.11] | 0.114 | 0.303 | 0.12 | -0.42 [-1.03, 0.20] | 0.98 [–0.73, 2.70] |
|  |  | 3000 | -0.55 [-0.80, -0.14]* | 0.013 | 0.036 | -0.30 [-0.69, 0.23] | 0.259 | 0.446 | 0.25 | -0.28 [-0.86, 0.31] | 0.68 [–0.98, 2.34] |
| %V̇O_2max_ | Spearman | 100 | -0.29 [-0.66, 0.19] | 0.212 | 0.313 | -0.31 [-0.71, 0.23] | 0.240 | 0.446 | -0.02 | -0.21 [-0.59, 0.17] | 0.34 [–0.51, 1.19] |
|  |  | 1000 | -0.07 [-0.51, 0.40] | 0.777 | 0.824 | -0.05 [-0.54, 0.47] | 0.854 | 0.882 | 0.02 | -0.10 [-0.60, 0.40] | –0.30 [–1.44, 0.84] |
|  |  | 2000 | +0.17 [-0.31, 0.58] | 0.486 | 0.599 | +0.07 [-0.45, 0.56] | 0.787 | 0.868 | -0.09 | +0.09 [-0.33, 0.52] | 0.13 [–0.85, 1.10] |
|  |  | 3000 | +0.17 [-0.31, 0.58] | 0.470 | 0.585 | +0.18 [-0.36, 0.63] | 0.506 | 0.697 | 0.01 | +0.09 [-0.31, 0.50] | –0.06 [–0.99, 0.88] |
| MFO | Spearman | 100 | -0.22 [-0.61, 0.26] | 0.359 | 0.476 | +0.14 [-0.39, 0.61] | 0.594 | 0.697 | 0.36 | +0.03 [-0.39, 0.44] | 0.22 [–2.09, 2.54] |
|  |  | 1000 | +0.50 [0.06, 0.78] | 0.024 | 0.059 | +0.51 [0.01, 0.81] | 0.041 | 0.147 | 0.01 | +0.48 [0.00, 0.95]* | 0.96 [–1.62, 3.55] |
|  |  | 2000 | +0.73 [0.41, 0.89]** | < 0.001 | 0.002 | +0.71 [0.31, 0.89]* | 0.002 | 0.014 | -0.02 | +0.61 [0.27, 0.94]** | –0.38 [–2.22, 1.46] |
|  |  | 3000 | +0.75 [0.45, 0.90]** | < 0.001 | 0.001 | +0.74 [0.37, 0.91]** | 0.001 | 0.009 | -0.01 | +0.61 [0.31, 0.92]*** | –0.61 [–2.28, 1.06] |
| ċLa_max_ | Spearman | 100 | +0.73 [0.42, 0.89]** | < 0.001 | 0.002 | +0.50 [-0.01, 0.80] | 0.049 | 0.155 | -0.23 | +0.54 [0.08, 1.01]* | 1.07 [–2.89, 5.03] |
|  |  | 1000 | +0.03 [-0.43, 0.47] | 0.915 | 0.922 | +0.29 [-0.26, 0.69] | 0.279 | 0.446 | 0.26 | +0.37 [-0.30, 1.03] | 0.13 [–5.62, 5.89] |
|  |  | 2000 | -0.41 [-0.73, 0.05] | 0.072 | 0.140 | -0.06 [-0.55, 0.46] | 0.820 | 0.875 | 0.35 | -0.04 [-0.62, 0.55] | 1.46 [–3.56, 6.48] |
|  |  | 3000 | -0.50 [-0.78, -0.06] | 0.024 | 0.059 | -0.17 [-0.62, 0.37] | 0.528 | 0.697 | 0.33 | -0.16 [-0.72, 0.40] | 1.18 [–3.62, 5.98] |
| La_RT_ | Pearson | 100 | +0.68 [0.33, 0.87]** | 0.001 | 0.006 | +0.30 [-0.25, 0.71] | 0.273 | 0.446 | -0.38 | +0.23 [-0.37, 0.82] | –0.75 [–2.48, 0.97] |
|  |  | 1000 | -0.07 [-0.50, 0.40] | 0.789 | 0.824 | +0.19 [-0.35, 0.64] | 0.487 | 0.697 | 0.26 | +0.15 [-0.75, 1.05] | –1.37 [–3.92, 1.18] |
|  |  | 2000 | -0.31 [-0.67, 0.16] | 0.189 | 0.293 | +0.15 [-0.39, 0.62] | 0.581 | 0.697 | 0.47 | +0.16 [-0.65, 0.97] | –0.64 [–3.00, 1.73] |
|  |  | 3000 | -0.48 [-0.77, -0.04] | 0.036 | 0.080 | -0.03 [-0.54, 0.49] | 0.909 | 0.909 | 0.45 | -0.03 [-0.77, 0.71] | 0.15 [–2.04, 2.34] |
| D' | Pearson | 100 | +0.69 [0.36, 0.87]** | 0.001 | 0.004 | +0.55 [0.07, 0.82] | 0.029 | 0.116 | -0.15 | +0.37 [-0.04, 0.77] | –0.60 [–1.76, 0.55] |
|  |  | 1000 | +0.10 [-0.36, 0.52] | 0.681 | 0.762 | +0.23 [-0.30, 0.65] | 0.395 | 0.602 | 0.13 | +0.35 [-0.28, 0.98] | 0.33 [–1.53, 2.19] |
|  |  | 2000 | -0.39 [-0.71, 0.06] | 0.088 | 0.156 | -0.16 [-0.61, 0.36] | 0.545 | 0.697 | 0.23 | -0.17 [-0.76, 0.42] | 0.23 [–1.50, 1.96] |
|  |  | 3000 | -0.61 [-0.83, -0.22]* | 0.005 | 0.016 | -0.38 [-0.74, 0.14] | 0.148 | 0.338 | 0.23 | -0.40 [-0.91, 0.10] | 0.18 [–1.31, 1.67] |
| ASR | Spearman | 100 | +0.55 [0.13, 0.81]* | 0.011 | 0.032 | +0.14 [-0.40, 0.60] | 0.610 | 0.697 | -0.42 | +0.20 [-0.33, 0.73] | 1.22 [–3.33, 5.77] |
|  |  | 1000 | -0.40 [-0.72, 0.07] | 0.084 | 0.151 | -0.36 [-0.73, 0.18] | 0.172 | 0.345 | 0.04 | -0.44 [-1.10, 0.21] | 1.77 [–3.80, 7.34] |
|  |  | 2000 | -0.72 [-0.88, -0.39]** | < 0.001 | 0.002 | -0.59 [-0.84, -0.11] | 0.017 | 0.076 | 0.13 | -0.63 [-1.12, -0.14]* | 2.26 [–1.81, 6.34] |
|  |  | 3000 | -0.85 [-0.94, -0.65]*** | < 0.001 | < 0.001 | -0.79 [-0.93, -0.47]** | < 0.001 | 0.005 | 0.06 | -0.83 [-1.20, -0.46]*** | 1.65 [–1.45, 4.76] |

ċLa_max_ = maximal lactate accumulation rate; La_RT_ = maximal post–exercise lactate concentration following the ramp test; MFO = maximal fat oxidation rate; RE = running economy (at maximal lactate steady–state); V̇O_2max_ = maximal oxygen uptake; %V̇O_2max_ = fractional utilization of maximal oxygen uptake (at maximal lactate steady–state); p = unadjusted probability value; q = p-value adjusted using the Benjamini–Hochberg false-discovery-rate procedure; Δ r = change in correlation coefficient.
